# Finding the right words: Articulating the value of mental health promotion. A focus group study

**DOI:** 10.1002/jcop.22801

**Published:** 2022-01-20

**Authors:** Johanna Cresswell‐Smith, Nonni Mäkikärki, Kaija Appelqvist‐Schmidlechner, Kristian Wahlbeck

**Affiliations:** ^1^ Equality Unit, Mental Health Team Finnish Institute for Health and Welfare (THL) Helsinki Finland; ^2^ Pro‐Lapinlahti Association Helsinki Finland; ^3^ Mieli, Mental Health Finland Finnish Institute for Health and Welfare (THL) Helsinki Finland

**Keywords:** mental health determinants, mental health impact assessment, mental health promotion, mental wellbeing, population mental health

## Abstract

The Lapinlahti Hospital initiative in Helsinki has transformed a disused psychiatric hospital into an open site for mental health promotion. The current study uses qualitative methods to explore how the initiative may promote population mental health. The phenomenological study comprised of data from 7 focus group including 28 participants. Resulting data were thematically analysed to articulate how the initiative supports mental wellbeing in different ways. Mental health benefits were categorized into three themes; mental health value, civil values and common values which were comprised of nine subthemes; paradigm shift, social inclusion, personal meaning, regeneration, ambience, stigma, sustainability, democracy and environment. Mental health promotion emphasises the impact of daily environments in which people live their lives. Results from this study support the use of broad based actions which promote different components of mental wellbeing simultaneously. Psychiatric hospitals may offer historically meaningful sites for such actions.

## 1 INTRODUCTION

Mental health promotion strives to support population wellbeing by fostering individual psychological capabilities (Jané‐Llopis et al., 2005; Kobau et al., 2011). Built on assumptions linked to positive psychology which views mental health as a valuable resource, mental health promotion moves away from the notion that mental health simply means the absence of mental illness (Huppert & Cooper, 2014; WHO, 2004). Public health approaches acknowledge population level benefits of these actions (Jané‐Llopis et al., 2005), which have been reported in terms of health, as well as social and economic outcomes (Jané‐Llopis et al., 2011; Knapp et al. 2011; Chida & Steptoe, 2008; Le et al., 2021).

Although the benefits of positive approaches to mental health have been recognised, a universal definition is yet to be formulated (Dodge et al., 2012; Vaillant 2012). Definitions tend to go by different names despite delineating similar concepts (Salvador‐Carulla et al. 2014), with many of them taking a multidimensional approach (Huppert, 2014; Linton et al., 2016). Definitions of *subjective wellbeing* for example, build on domains such as evaluative wellbeing (referring to overall satisfaction with life), hedonic wellbeing (including positive and negative affect), and the eudemonic wellbeing (relating to how meaningful life is) (Diener, 1984; Dolan et al., 2011; Kapteyn et al., 2015). Ryff's model of *Psychological wellbeing* on the other hand is conceptualised as including six different components related to positive functioning, namely autonomy, environmental mastery, personal growth, purpose in life, positive relations with others and self‐acceptance (Ryff, 1989), while Seligman's *PERMA model* emphasises Positive Emotion, Engagement, Relationships, Meaning and Accomplishment (Seligman, 2011).

Despite their differences, the multidimensionality of these approaches provide a good idea of the breadth required for mental health promotion (Cresswell‐Smith et al., 2019; Goodman et al. 2018; Lambert et al., 2015). On an individual level this may involve interventions which improve an individual's mental health literacy, mental health awareness, coping skills or resilience, (Melbourne Charter for Promoting Mental Health, 2008; Saxena et al. 2006a). Population based approaches on the other hand emphasise broader actions which nurture the day‐to‐day environments in which people live their lives (Shim et al., 2014; Silva et al., 2016) for example by ameliorating the wider determinants of mental health, and building mentally healthy conditions in society (Allen et al., 2014; Barry 2008; Shim et al., 2014; Silva et al., 2016). Both approaches are founded on principles such as protecting the positive, and encouraging opportunities for flourishing, that is; ‘feeling good and functioning effectively’ (Hone et al., 2014; Keyes, 2002). Levels of flourishing has been found to vary across countries and cultures (Huppert & So, 2013), which may point to effects of different policy priorities, and to what extent societies are able to cultivate mental wellbeing. For the sake of simplicity, and as detailed definition of individual components is not the focus of this study, the current study will use the term *‘mental wellbeing’* to bridge these different concepts whilst acknowledging positively orientated multidimensional approach.

The Mental Wellbeing Impact Assessment Framework (MWIA) describes protective factors to population mental wellbeing including opportunities for having control over one's life, for pursuing one's goals, and having autonomy and self‐efficacy in life (Cooke et al., 2011). Furthermore, improving participation and social inclusion via leisure and cultural actions, volunteering and other forms of collective and civic engagement (Jenkinson et al., 2013; Jones et al., 2013) is highlighted as beneficial, as is encouraging resilience, and making use of community assets (Herrman et al., 2011). The importance of building community connectedness has also been noted in this light (Annor & Allen, 2009; Castillo et al., 2019; Friedli, 2000), as has the influence of *‘attachment to place’*. That is, promoting mental health by taking into account *where* such actions take place (Macintyre et al., 2002; Norris et al., 2008). A recent study placed particular importance on historical and natural green landscapes in their role in promoting mental health in urban contexts (Hosseini et al., 2021), with others echoing the need for more ethical approaches within urban development (Högström & Philo 2020).

An example of symbolic heritage attached to *place* can be found in connection to old psychiatric hospitals in terms of their cultural and historical significance (Engstrom 2008). Old psychiatric hospitals are physical reminders of what how we viewed mental health through the ages, (Cookson, 2012) allowing us take a critical look at the past, while generating insights into contemporary approaches and what needs to be discussed today (Hess & Majerus, 2011; Hilton, 2018). There are several accounts of former psychiatric hospitals being repurposed. Some of these step away from their original purpose completely by actively severing all ties to the past (e.g., into new housing developments), while others preserve their links to their past, for example with hospital museums containing historical artefacts and other collections such as art exhibitions pertaining to mental health. Bethlem Hospital in London (Northwood, 2014) has had a museum on its premises since 1970 generating a deeper understanding for humanity and the cultural mechanisms around mental health stigma and social exclusion (Topp et al. 2020; Tweed & Sutherland, 2007). However, many sites remain unused and derelict (Kearns & Moon, 2015). Although the process of repurposing has been reported elsewhere, less is known about how these actions may contribute towards promotion of population mental health.

The focus of the current study is to explore how initiatives at a repurposed psychiatric hospital can be used for promoting population mental health. The study setting is a contemporary initiative based at the former Lapinlahti Hospital in Helsinki. Commissioned by the Grand Duke of Finland, the historic psychiatric hospital opened its doors in 1841 and was the flagship of psychiatric care in Finland until 2008 when, in line with the deinstitutionalisation movement, psychiatric inpatient care was transferred to general hospital settings, instead of being centralised to stand‐alone psychiatric hospitals (Pylkkänen, 2012). After several years of dereliction, the former hospital has recently been transformed into a site for community activities built along five principles namely innovative working culture, training, working and volunteering opportunities, mental health and wellbeing, cultural heritage and social inclusion (Helsinki's 2021). The initiative opens up the historical landmark to the public, protects and preserves the building and its surrounding parkland, while providing a host of activities which promote mental health both directly and indirectly. Measuring the impact of such broad‐based actions requires innovative approaches (Moore et al., 2018). The MWIA framework provides a multifaceted framework for assessing these actions, which may not lend themselves to assessment via distinct measures or indicators (Cooke et al., 2011).

## 2 MATERIALS AND METHODS

### 2.1 Study setting

The current study used qualitative methodology based on a phenomenological approach (Neubauer et al., 2019) to explore how mental wellbeing may be promoted at the former Lapinlahti Hospital. The context of these actions is an important factor to consider. As mentioned above, Lapinlahti hospital has been repurposed from a clinical setting to a centre promoting mental wellbeing. Following progression of the deinstitutionalisation movement, the hospital closed its doors in 2008 and stood empty for almost 10 years. In 2015, Mental Health Finland (www.mieli.fi/en), a mental health NGO, developed a new concept for the hospital site in collaboration with sister organisations such as the Pro‐Lapinlahti Association, in the form of citizen‐led mental health promotion actions. These include activities such as workshops, lectures, art and cultural activities, chess or board game groups, as well as a café, second hand and handicraft stores and a sauna. The site is located in the centre of Helsinki, enclosed in an expanse of parkland within a scenic bay. The initiative also has allotment gardening plots, and arranges various nature orientated groups such as bird watching, planting, and outdoor and conservation activities and historical tours. The centre hosts group activities such as low threshold coffee gatherings aiming to engage people with lived experience of mental health difficulties, or otherwise in a potentially marginalised position, and has an ‘open living room’ where anyone can come and spend some informal time socialising. The arts are an important theme running throughout, and visitors can view various constellations along the corridors, as well as visiting a mental health museum depicting both the history of psychiatry and contemporary exhibitions relating to mental health. Furthermore, office space is available for therapists and micro‐enterprises who engage in actions which can be considered to support mental health. Activities are open for anyone and are predominantly free of charge, with the exception of therapy sessions and various items for sale in the café, or second‐hand shop. During data collection, the initiative was under threat of discontinuation, with the City of Helsinki advancing with its intention to sell the premises.

### 2.2 Data‐collection

Participants were primarily recruited via convenience sampling via open invitations to take part in a focus group exploring how the Lapinlahti initiative impacts on mental wellbeing. A brief summary of how results were going to be used was also included in the invitation, i.e., that it will be part of a PhD study, and used to inform proposals delineating the future of the Lapinlahti initiative. Invitations were placed on the Lapinlahti Hospital notice board and spread through social media (Facebook) in March 2019. Participants were also encouraged to spread the invitation to others, therefore, a certain level of snowball sampling was also employed. Participation was welcomed from a broad range of individuals including visitors, employees, tenants, volunteers and trainees on supported employment placements.

Focus groups were conducted in a meeting room at the Lapinlahti site between March 2019 and April 2019 by a moderator and an assistant, roles shared by authors JCS and NM who were present in all focus groups, KW also participated in a focus group. A semi‐structured interview guide was used to structure the focus groups based on the Dynamic Model of Mental Wellbeing (Cooke et al., 2011), looking at mental wellbeing in different population groups, the wider determinants of mental wellbeing and protective factors for mental wellbeing. The interview guide was refined in a meeting between researchers and study site representatives. Focus groups methodology is particularly useful for generating discussion and richer data by capitalising on the interaction between participants (Kitzinger, 1995).

The interview guide consisted of a slide presentation and provided a 30 min introduction outlining the general process of the focus group, the informed consent procedure as well as providing definitions to support conceptual clarity around mental health ill health and mental wellbeing including differences between these. Clarity was aided using a visual diagram of the Dynamic Model of Mental Wellbeing wheel (Cooke et al., 2011) and outlining all terminology including wider determinants of mental wellbeing as well as the protective factors for mental health. In addition to the presentation, participants had corresponding printed material at hand for reference. Participants were also asked to fill in very brief survey on background information in terms of age, gender and role at the Lapinlahti Hospital. Participants were offered refreshments and time to ‘warm up’ into discussions during the introduction presentation.

Seven focus groups were held including a total of 28 participants. The smallest group consisted of two participants (plus moderator and assistant moderator) and the largest focus group consisted of seven participants.

The focus group interviews were held in three main languages used in Lapinlahti (comprising of one Swedish group, one English group and five Finnish groups). Focus group lengths ranged from 35 to 55 min, with an average length of 47 min. We asked participants to discuss thoughts around five main questions;

(1) What population groups are particularly affected by actions at the Lapinlahti initiative?
(2) Does the Lapinlahti initiative impact the wider determinants of mental wellbeing?
(3) How does the Lapinlahti initiative impact on the protective factors for mental wellbeing? Is any one of these more relevant than the other?
(4) Are there any negative impacts?
(5) How has the Lapinlahti initiative impacted your mental health?

The moderator's task was to present the slide show, lead and maintain the focus of the interview, making sure all participants contributed as they wished, and to facilitate discussion between participants. The assistant observed dynamics in the group and looked after practical aspects of the discussion.

All participants gave written informed consent in their language, agreeing to data being recorded, transcribed and translated. Anonymity was explained and affirmed, as was the possibility for withdrawing from the study at any time. Data was collected based on principles of informed consent, following ethical principles of research in accordance with the Finnish National Board on Research Integrity TENK guidelines (Finnish National Board on Research Integrity TENK guidelines, 2019).

### 2.3 Data‐analysis

Focus groups were digitally recorded and transcribed verbatim and all respondents were allocated a unique anonymized code. Transcriptions totalling 120 pages containing 35,774 words (Times New Roman, size 11, 1.5 line spacing) were imported into NVivo 12 Pro for Windows qualitative software programme (NVivo, 2018). Excerpts used in this report were further translated into English.

Following this, transcriptions were analysed using thematic analysis looking at experiences and meanings generated from the questions outlined above. This approach follows a realist approach looking not so much at who said what, but rather at what was said (Braun & Clarke, 2006) about the ways in which the initiative promotes mental health in different ways. The iterative and reflective analyses followed an inductive, data driven approach on the semantic level (Boyatzis, 1998; Graneheim et al., 2017), following six steps as outlined by Braun and Clarke, with particular focus on qualitative criteria in relation to trustworthiness (Braun & Clarke, 2006).

In the first step, transcriptions were read several times to gain a solid familiarity with the content. Following this, the data was reread although this time making preliminary note of citations depicting meanings in relation to how mental wellbeing is promoted at the Lapinlahti hospital, picking out those which emerged in a particularly profound or repetitive pattern. These notes constituted the first step in terms of defining what thematic topics were brought up in conversations. In a second step, initial codes were named describing central topics, and data was reread again with these initial codes in mind. The third step involved these codes to be collapsed into larger themes and renamed accordingly. Codes were collapsed and renamed if they were deemed to describe similar topics or if they could be described in a clearer manner. The resulting themes depicted the different characteristics or ways in which the Lapinlahti initiative may promote the mental wellbeing of its visitors, workers and volunteers. Themes which were found not to relate to promotion of mental wellbeing, for example those with a more advocative tone centred around the aforementioned risk of the closure were omitted.

**Figure 1:**
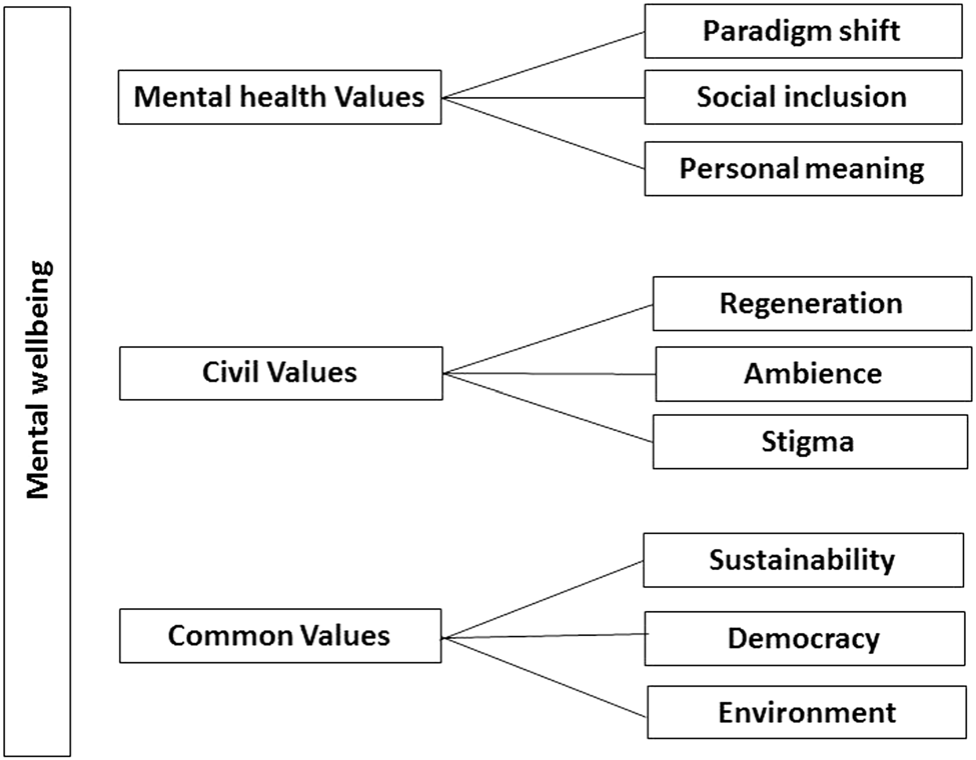
Themes and subthemes derived from focus groups

In the fourth step, the resulting themes were then further collated and reviewed in relation to the full data set, paying particular attention to the fit and validity of each theme. The fifth step involved clustering the final nine themes under three broad thematic headings, forming the final set of themes and subthemes (see Figure 1). The sixth and final step involved writing up the study and making sense of the results in relation to the current and previous studies.

One author (JCS) coded all transcriptions, and to support credibility and validity (Leung, 2015), all themes and subthemes were further scrutinized by NM who attended all focus groups and read and reread all transcriptions. All citations and subthemes were explored and discussed and decisions in terms of the final selection of citations to be included in the paper were made together. Themes were reviewed by all co‐authors who had full access to the data. Discrepancies in definitions, categorizations or interpretations were resolved via discussion until consensus was reached.

## 3 RESULTS

Participants were predominantly female (68%), aged between 30 and 69 years of age (61%), and were mainly involved in the Lapinlahti initiative as a visitor (43%). There was representation from all roles including visitors, volunteers, employees and trainees. One participant ticked the ‘other role’ box and defined themselves as a peer group leader in the open answer space.

Although the premise for the discussion was broad, the interview guide based on the MWIA framework provided a clear structure and allowed the discussion to hone into broader determinants and protective factors for mental health and wellbeing. Three broad themes were derived from the data: mental health value, civil values; and common values. The mental health value theme depicts how the initiative influences aspects related to how mental health is viewed and addressed. The civil values theme on the other hand describe different ways in which the initiative engages community, or civil components. Thirdly, the common values theme reflects broader more macro level effects impacted by the initiative. Each theme includes three further subthemes depicting more specific ways in which actions at the Lapinlahti initiative promotes mental wellbeing.

### 3.1 Mental health values

In this theme participants discussed aspects relating to subthemes of paradigm shift, social inclusion and personal meaning. These subthemes related more directly to participants own mental health, or the mental health of people involved in the actions at the Lapinlahti hospital. Within the paradigm shift subtheme, participants discussed the importance of recognising existing strengths instead of taking a problem‐centred view of mental health. Participants reported Lapinlahti as a site reflecting the current paradigm shift within mental health, describing actions of the Lapinlahti initiative as a living example of changes in the way we talk about mental wellbeing, describing the site as “a living room for all” meaning an open space where everyone is welcome to spend time.

Participants talked about Lapinlahti former hospital being a place for everyone, with no need for diagnoses therefore normalising mental health difficulties and viewing them as something which can affect anyone. They also discussed intergenerational changes in the concept of mental health, and how the younger generation talks about mental health in a different light which also reflects the current paradigm shift.

*“For me it is really nice that this is so clearly, or at least in my head has been contoured into …a centre for wellbeing…that there is no such stigma as in the past…the more you get away from [the assumption] that you have to have some sort of mental disorder…or that ‘oh you must be really unwell to be here’. That we could get to a place where [we think] ‘hey this [place] is for everyone!” (303/visitor)*

A core function of actions at the Lapinlahti is to support social inclusion of people in marginalised positions in society, something discussed by participants in the focus groups. Participants described benefits of having the opportunities to try out skills in a safe environment, and of being accepted as you are, also when not feeling at ones best. Although only two participants were from this category i.e. currently involved in the trainee programme at Lapinlahti, many were aware of the supported employment training opportunities at the hospital and recognised their importance.

*”…[at Lapinlahti] there are opportunities of achieving a lot of strengthening experiences and like…that you can get involved be included, having influence, trying things, learning things…” (101/employee)*

Furthermore, participants discussed long‐term benefits of being able to access activities which promote social inclusion, describing opportunities at Lapinlahti as having a positive springboard effect, generating a sense of hope and resilience for the future.

*”And I believe… in my potential in a totally different way compared to before, like, that I can go elsewhere after this” (101/employee)*

The personal meaning subtheme was derived from discussions around valuable and meaningful experiences brought about by the Lapinlahti initiative. Participants expressed these as a sense of mutual understanding, articulating a sense of catharsis which had a profound impression on mental wellbeing. Participants also talked about these experiences of personal meaning as generating peace of mind, giving strength and initiating deeper understandings and thought processes

*”…I do think that this is for many the kind of place which increases [feelings of] meaningfulness…there is kind of, important values [for many] which are achieved for example [through] conservation…history, historic conservation, it gives a certain value to the initiative” (502/employee)*

### 3.2 Civil values

The civil values theme related to change brought on by civil society actions at the Lapinlahti site. This change was discussed in a historical sense and as a shift in purpose at the Lapinlahti Hospital. As well as being illustrated in the regeneration subtheme, this shift was also reflected in terms of how the atmosphere or ambience surrounding the hospital has influenced broader aspects such as stigma associated with both the hospital and mental health in general.

Within the subtheme of *regeneration*, participants reflected on the mental health impact of re‐purposing the old psychiatric hospital from a closed institution to an open centre for mental wellbeing. They reflected on how this has placed it on the map and activated new ways of promoting mental health. Participants also related the historical value of the hospital and how it can be used to open up discussions and thoughts on mental health today. Many who were involved developing or otherwise active in the initiative talked about positive feelings connected to co‐developing a new purpose for the hospital site.

*”When I involved myself with this initiative I felt it was absolutely lovely that I could be here helping to breathe new life into this place!….lets air all of the heavy things away!” (501/volunteer)*

Participants also referred to the ambience which has grown from the actions at the Lapinlahti site, and articulated mental health benefits of such an atmosphere. This ambience was described in terms of Lapinlahti being a place to be with no preconceived meanings, a place where one can simply be, even if having a bad day. They also talked about benefits of and unifying nature of the positive and accepting ambience of Lapinlahti Hospital including the freedom to engage with others as much or as little as they wished.

*“Like there is a lot of empathy, and like the approach is amazing. So actually runs in harmony.” (701/visitor)*

Opening up the hospital site to the public was also felt to have a positive impact on stigma associated with mental health difficulties, both in terms of opening up opportunities for awareness‐raising as well as allowing people have contact with people from different walks of life in a very equitable way.

*“*…*as this is an open place and [anyone] can come here and do things…participate then it supports a kind of anti‐stigma work in a way that wellbeing is a good thing and it does not mean illness but promoting wellbeing.” (601/employee)*

The hospital in itself as a former psychiatric institution was referred to as a conduit for this stigma reduction effect, describing positive effects of the site being open for everyone and in terms of meanings stemming from the cultural heritage associated with the location itself (Table 1).

**Table 1:** General demographic characteristics of participants

	Frequency	Percentage
Gender		
Female	19	68
Male	9	32
Total	28	100
Age		
18–29	8	29
30–62	17	61
63–79	3	11
Total	28	100
Role		
Visitor	12	43
Volunteer	4	14
Employee	9	32
Trainee/intern	2	7
Other	1	4
Total	28	100

### 3.3 Common values

The common values theme reflects general macro‐level societal values which the Lapinlahti initiative taps into. These are further divided into three subthemes namely sustainability, democracy and environment. Sustainability was interpreted as relating aspects relating to access (to activities), as well as benefits stemming from them. In contrast, the democracy subtheme was depicted in terms of everyone in society having equal access to actions which promote mental health. Finally, the environment subtheme related to environmental benefits of the initiative.

Participants acknowledged how actions at the Lapinlahti site reflected an ethos of sustainability, shifting focus away from purely economic benefit towards social and individual benefits. This was a very strong theme within the focus groups.

*”…[if] people [get the opportunity to] arrange different actions which support their spiritual wellbeing, then I don't know in what way we could calculate the [profitability] of this. But if I think about the group that I run, and think about how many trips to the healthcare centre it has saved…” (401/other – peer group leader)*

Participants also pondered the monetary value of actions along this theme, of how much money is saved by allowing the population access to such an environment, but also in terms of how money is not a barrier to taking advantage of actions at Lapinlahti. Participants valued the noncommercial ethos of the Lapinlahti initiative and opportunities for engaging in life‐long learning and volunteering.

*“…and that is what Lapinlahti has realised and is doing very well with this kind of very modern education, lifestyle education and music and meeting and learning from each other and everything is done indirectly” (704/visitor)*

The subtheme of democracy related to how actions were available for all of society, but also in terms of the unifying quality of the Lapinlahti initiative. Participants describe the richness and value of different population groups mixing, generating a sense of openness and tolerance.

“*In my opinion it is good that there are different kinds of open activities…it is exactly the openness that everyone can come… and it mixes like people in different life‐situations and …and that is the richness that [people with experience of mental health difficulty] don't just sit [with each other] but you can [interact] with anyone at all…and like in a way we are all in the same boat.” (402/employee)*

It was felt important that a grand location such as Lapinlahti should be available to all sectors of society, particularly those who do not otherwise have the opportunity to enjoy such sights. Participants highlighted that it is only fair that all walks of life have access to valuable areas within a city, not only those who are well off or otherwise have access to beautiful surroundings.

The focus group participants did not have difficulty with articulating positive mental health benefits stemming from the scenic environment surrounding Lapinlahti site, particularly in relation to the unique opportunity of accessing nature in the middle of the city. Participants also related this to the original purposes of the area as an area to soothe the mind and the importance of having easy access to positive environments’ also today.

*”…this is an environment which looks after the soul, partially in a historical sense …but also [contemporary]. It is something which creates a wish to want to be here…I mean if this was a concrete town I don't think people would make the journey, it's the environment which makes people want to come here…” (201/visitor)*

The citations illustrate how mental well‐being was derived from the Lapinlahti initiative in different direct and more indirect ways. The sample represented different types of people involved in the initiative including the target group i.e. ordinary citizens regardless of background. The focus group discussions produced subthemes which represented different ways in which activities stimulate and promote mental health, which were clustered into three main themes depicting three broader (value) levels which the activities influence (see Figure 1).

## 4 DISCUSSION

The Lapinlahti initiative promotes mental health by merging broad actions based on cultural heritage, civil society as well as principles of sustainable urban development. Themes and subthemes derived from the study are useful ways of concretising how these actions support population mental wellbeing.

### 4.1 A sense of meaning

Several subthemes in this study made reference to a sense of meaning highlighted by eudaimonic approaches to mental wellbeing such as Ryff's model of psychological wellbeing (Ryff, 1989), and Seligman's PERMA model (Seligman, 2011). Participants in the current study reflected on the importance of having access to opportunities for experiencing a meaningful daily life. Context and place played an important part in this regard, with subthemes of *regeneration* and *ambience* characterising how a sense of meaning could be derived from repurposing Lapinlahti Hospital from its original purpose as a psychiatric hospital to a contemporary centre for mental wellbeing, and stimulating new ways of co‐developing positive actions. Studies indicate that people with high levels of eudaimonic wellbeing tend to emphasise personal growth, and the value of achieving positive insights in life (Bauer et al., 2008).

History can provide us with important insights both from a practice and sociocultural perspective (Beer, 2009; Houston, 2019). Within the *regeneration* subtheme, participants’ related meaning in connection with the hospital's historical significance on both an individual and societal level. Re‐purposing old psychiatric hospital sites has been done in different ways in around the world, many of which build on the historical meanings derived from the site itself. Kearns and Moon (2015) describe the juxtaposition of the origins of the ‘asylum’ which in the eighteenth century seen as a safe‐haven encompassing the prospect of recovery, later developing into large institutions which often fostered mistreatment and abuse (Kearns & Moon, 2015, p 3). Participants in the current study acknowledged the problematic past, and how it can be used to create something new “*… it was absolutely lovely that I could be here helping to breathe new life into this place!”*. Improved accessibility to old psychiatric sites have produced positive results also elsewhere, for example by reducing mental health related stigma (Högström & Philo 2020).

Meaning was also attributed to the unique atmosphere held at the Lapinlahti Hospital. Subthemes describing the *ambience* or atmosphere at the Lapinlahti site made references to a sense of tolerance, understanding and acceptance. Actions were described as having a low threshold, and participants related this to the welcoming nature of workers and volunteers at the initiative. The multisector approach largely driven by citizen‐led NGO actions may have contributed to this atmosphere. Previous studies report NGO workers to have more positive attitudes, and reduced tendency to socially distance themselves from people with mental health difficulties in comparison to healthcare workers (Rose et al., 2018). Other studies also underline benefits of approachability, flexibility, partnership working, and user involvement often found within NGO's, characteristics which can be especially useful for engaging with hard to reach groups (Flanagan & Hancock, 2010). Civil society is generally well placed to bring mental health promotion into the daily lives of the population, rather than being something which only the social and healthcare sector is involved in (Cresswell‐Smith et al., 2020).

### 4.2 Contemporary views of mental health

Subthemes of *paradigm shift*, *social inclusion* and *stigma* all depict prevailing and contemporary views on mental health. Participants described the Lapinlahti Hospital initiative as a living example of the ongoing paradigm shift. This paradigm shift views mental health as more than just ill‐health and acknowledges mental health difficulties as something which can affect anyone. In this line, participants described the new initiative at Lapinlahti Hospital as a place for everyone, regardless of health status of diagnoses.

Opportunities for being accepted as you are lies at the heart of this paradigm shift, following principles of social inclusion. Participants in the current study emphasized the need for acceptance, even when you are not feeling at your best. These insights were discussed in the context of supported employment placements provided at Lapinlahti Hospital initiative. On a policy level, providing opportunities for everyone to enjoy ‘full and effective participation and inclusion’ (e.g., via supported employment placement) is a fundamental part of the UN Convention on the Rights of Persons with Disabilities, and is particularly important in relation to disadvantaged communities (Cruwys et al., 2014; UN General Assembly, 2007). On a more individual level social capital has been cited as an important determinant of psychological wellbeing (Nieminen et al., 2010).

Participants in the current study made multiple references to the destructive nature of stigma, despite viewing mental health difficulties as something which can affect anyone. Making references to its historical past, participants described the re‐purposed site as initiating new dialogues around mental health, as well as generating new opportunities for social contact with people from all walks of life both of which were considered to be useful for reducing mental health related stigma. Mental health related stigma has been described as a complex phenomenon with worse consequences than the mental health conditions themselves (Corrigan & Watson, 2002). Previous studies have found social contacts to reduce the ‘us’ and ‘them’ mechanisms which fuel mental health related stigma and social exclusion (Helmus et al., 2019; Thornicroft et al., 2016). Opportunities for recognising mental health difficulties as something which can affecting our ‘own group’ are therefore likely to some way to reduce mental health related stigma in our societies (Schomerus et al., 2013). A sense of meaning was also derived from being part of the new initiative, making new memories for the place.

### 4.3 Determinants of mental wellbeing

Population mental health and wellbeing depend on the circumstances of our daily lives (Allen et al., 2014). This rings true also in terms of the context of mental health promotion (Barry, 2009; Saxena et al. 2006a). Participants in the current study, made references to the importance of key determinants of mental health within the subthemes of *sustainability, democracy and environment* highlighting the need for economically accessible and environmentally sustainable activities. Removing economic barriers to meaningful activities is important for promoting population mental health, as both physical and mental health outcomes tend to follow a social gradient with in higher socioeconomic groups being in more favourable positions (Marmot, 2010; Marmot & Bell, 2012).

The subtheme of *democracy* followed a similar but broader line, highlighting the need for positive activities to be available for everyone in society. Participants expressed the need for celebrated built and natural environments to be accessible for everyone in society, not only the (financially) privileged few which may represent an increased awareness of income inequality (Keeley, 2015) or be a reflection of an overall trend towards sustainable lifestyles based on values which are not solely monetarily led. The importance of equitable access to actions which support positive mental health has also been argued by Wise and Sainsbury who coined democracy as 'forgotten determinant of mental health', associating increased democratic rights, participation, and autonomy with higher levels of mental wellbeing (Wise & Sainsbury, 2007).

The *environment* subtheme depicted mental health benefits of the natural surroundings encasing the Lapinlahti Hospital, resonating with a growing body of evidence indicating that urban green spaces have a protective effect on the mental health of both children and adults (Lee & Maheswaran 2011). Nature connectedness, or an individual's subjective sense of their relationship with the natural world, (Mayer & Frantz, 2004) has shown links to both hedonic and eudaimonic dimensions of mental wellbeing and levels of personal growth (Capaldi et al., 2014; Pritchard et al., 2020). Furthermore, engaging in activities characterised as purposeful and meaningful in the context of public green spaces were found to produce benefits to physical and mental health including hedonic and eudaimonic dimensions (Coventry et al., 2019). Links between the built environment and mental health are increasingly being recognised, although the potential for improving public mental health via urban development arguably requires more attention (McCay et al., 2017; Pfeiffer & Cloutier, 2016). Making use of existing cultural heritage sites for health promotion purposes could combine the interests of both public health and urban development sectors.

### 4.4 Implications and future directions

The Mental Health in All Policies approach (MHiAP) approach underscores the need for synergy across sectors for developing local or national initiatives which support mentally healthy societies Botezat et al., 2017). Calls for actively creating more wellbeing‐promoting societies have been made by scholars such as Slade, who also remind us that the objectives behind the New Economics Foundation Five Ways to Wellbeing (Aked et al., 2011): Connect; Be active; Take notice; Keep learning; and Give are all outwards‐looking' i.e. focusing on promoting engagement in the community and others (Slade, 2010).

Findings from the current study indicate that many important components of mental wellbeing can be reached simultaneously via broad community actions. It is worth noting that these conditions do not occur by themselves but rely on favourable conditions being actively cultivated. Population based approaches to mental health promotion are dependent on mental health being viewed as a public health issue (Purtle et al., 2020), a shift which appears to be underway (Forsman et al., 2015; Friedli and World Health OrganizationRegional Office for Europe ‎^2009^; Wahlbeck, 2015). Decision makers need to be able to make evidence informed policies to translate this shift into concrete actions. Scientific outcomes need to be easily accessible and be *“in the right format and at the right time”* (OECD 2020). The evidence base needs diverse and well‐matched methodologies, as traditional comparative effectiveness research may not always capture the complex nuances of broad‐based actions. The current study is an example of how this can be achieved.

### 4.5 Strengths and limitations of the study

The current study used qualitative methods to explore the meaning, breadth and importance of mental health promotion actions at Lapinlahti Hospital in Helsinki. This provides insights on how people interact with the built environment and places of historical, cultural and social significance. While the study took a unique approach looking at how actions for mental health promotion can be developed within a disused psychiatric hospital, it is important to acknowledge some limitations.

Firstly, although qualitative research methods lend themselves well to inductive development of new approaches and making sense of unique phenomena (Collins & Stockton, 2018), causal conclusions are not possible. The diversity of the participant sample is a particular strength, enabling collection of data in three different languages and different stakeholder groups. The sample represented different types of people involved in the initiative as well as the target group i.e. ordinary citizens regardless of background. Although the Lapinlahti hospital has its roots in patient care, no patients are currently being treated at the site, and patients’ experience of treatment was therefore not a part of this study. As the emphasis was on population mental health, and the ethos of the Lapinlahti initiative builds on the notion that everyone is welcome (making a particular point of not needing to disclose any diagnoses), a more universal approach was appropriate.

Although participants predominantly brought up positive aspects of the initiative at Lapinlahti Hospital, it is important to acknowledge that an old psychiatric hospital can also generate negative feelings, thoughts and memories. Psychiatric hospitals have throughout the ages have housed fear, human rights abuses and other traumatic experiences (Stone, 2008), and it is likely that anyone with difficult memories attached to the Lapinlahti Hospital did not attend the focus groups. Although question four in the current study approached this subject on superficially, further study using a more targeted recruitment approach is needed to explore this avenue in more depth.

The interview guide built on the MWIA framework to ensure participants had the same understanding of key concepts and used these terms in a consistent manner. It was deemed important that all participants received an introduction to the main concepts, as terminology around positive mental wellbeing has anecdotally been found to be frequently misinterpreted. Although the intention with this approach was to facilitate discussion and harmonise terminology, it is worth noting that use of the MWIA framework could also have had the undesired effect of steering participants towards specific concepts, rather than allowing participants to freely articulate their thoughts. Moderators were cognisant of these issues throughout and had prepared themselves to be actively involved in terms of clarification but also encouraging a free discussion.

On a positive note, and despite moderators’ initial concern in relation to how the interview guide and background material would be absorbed, it was quickly noted that participants did not have difficulty with terminology, or staying on the positive side of mental health. This could have been a product of the interview guide, or potentially due to the Lapinlahti initiative underlining this paradigm shift in all actions. The atmosphere within the focus groups was positive and discussions flowed naturally. All groups contained more talkative participants and less talkative participants, and moderators endeavoured to steer turn taking in a natural fashion without forcing answers. Separate specific analysis in terms of saturation was not included. Despite some focus groups being rather small this did not appear to limit richness of discussions.

It is worth bearing in mind that the context in which participants were interviewed may have impacted their discussions. Interviews were held during a process where the actions at the Lapinlahti Hospital was under threat of closure, which may have impacted results in several ways. Participants were recruited via convenience sampling, and although representation from all groups was achieved, it is likely that it included individuals who were especially eager to discuss benefits of the initiative, and may well have been motivated to use the focus groups as a way of advocating for continuation of the initiative. Although this type of motive may not necessarily have changed discussions in terms of content, citations which were interpreted as specifically defending or championing the initiative were omitted from analysis. Authors do however recognise the potential for overlap.

Finally, all authors with the exception of KA‐S have been involved in the Lapinlahti initiative in different ways, and this potential influence has been considered throughout the analysis and writing procedure. The intention was not explicitly to provide support for the Lapinlaht Hospital initiative, but rather to use it as an opportunity to explore how such open mental health promotion actions could impact population mental wellbeing. Despite these potential limitations, the current study provides valuable insights on mental health promotion in this context.

## 5 CONCLUSION

The current research study found the Lapinlahti Hospital initiative to produce broader benefits via its sustainable and democratic actions suitable for all population groups, as well as benefits relating more directly to mental health promotion as illustrated in the subthemes. All themes and subthemes build on the assumption that mental health is a resource which can be supported in the context of the daily environment. The results also line up with a MHiAP approach which highlights benefits of including mental health promotion in all sectors and on all levels.

The predominantly citizen‐led actions at the Lapinlahti Hospital produced an open and accepting atmosphere which may be particularly useful for this type of mental health promotion. Needless to say, endeavours for mental health promotion need to be part of a larger picture of health promotion and health services. Positively aligned approaches are not intended to replace or place less focus on the need for balanced mental health services.

Collaborative approaches are needed to include mental health promotion as an area of importance across all sectors. Making use of symbolic sites such as old psychiatric hospitals can utilise their unique historic value, and develop social, political and psychological significance.

## FUNDING INFORMATION

Johanna Cresswell‐Smith would like to thank Finska Läkaresällskapet for funding the opportunity to complete her PhD.

## CONFLICT OF INTERESTS

The authors declare the following financial interests which may be considered as potential conflict of interests:

Johanna Cresswell‐Smith, although not involved in the Lapinlahti project in an official capacity has run some workshops and events at the location. JCS is employed by the Finnish Institute for Health and Welfare (THL).

Nonni Mäkikärki was at the time of data gathering was not employed by the Lapinlahti initiative, but was part of the same community mental health network as Lapinlahti Hospital. During final steps of the article writing process, NM was employed as the Director of the Pro Lapinlahti Association, one of the main drivers of the Lapinlahti initiative.

Kristian Wahlbeck is the Director of Development at MIELI Mental Health Finland, the founding organisation of the Lapinlahti initiative, and is an active Member of the Board for Pro Lapinlahti Association and Lapinlahden Lähde Ltd, a not‐for‐profit social enterprise responsible for leasing office spaces within the Lapinlahti Hospital. KW has a dual affiliation as a Research Professor at the Mental Health Unit at the Finnish Association for Health and Welfare (THL).

Kaija Appelqvist‐Schmidlechner is employed by the Mental Health Unit at the Finnish Institute for Health and Welfare (THL) and declares no conflict of interest.

### PEER REVIEW

The peer review history for this article is available at https://publons.com/publon/10.1002/jcop.22801

## Data Availability

Research data are not shared.
